# Relationship Between G-Quadruplex Sequence Composition in Viruses and Their Hosts

**DOI:** 10.3390/molecules24101942

**Published:** 2019-05-20

**Authors:** Emilia Puig Lombardi, Arturo Londoño-Vallejo, Alain Nicolas

**Affiliations:** Institut Curie, PSL Research University, UMR3244 CNRS, 75248 Paris CEDEX 05, France; maria-emilia.puig-lombardi@curie.fr

**Keywords:** G-quadruplex, virus, eukaryotic hosts, Herpesviridae, genome evolution

## Abstract

A subset of guanine-rich nucleic acid sequences has the potential to fold into G-quadruplex (G4) secondary structures, which are functionally important for several biological processes, including genome stability and regulation of gene expression. Putative quadruplex sequences (PQSs) G_3+_N_1–7_G_3+_N_1–7_G_3+_N_1–7_G_3+_ are widely found in eukaryotic and prokaryotic genomes, but the base composition of the N_1-7_ loops is biased across species. Since the viruses partially hijack their hosts’ cellular machinery for proliferation, we examined the PQS motif size, loop length, and nucleotide compositions of 7370 viral genome assemblies and compared viral and host PQS motifs. We studied seven viral taxa infecting five distant eukaryotic hosts and created a resource providing a comprehensive view of the viral quadruplex motifs. Overall, short-looped PQSs are predominant and with a similar composition across viral taxonomic groups, albeit subtle trends emerge upon classification by hosts. Specifically, there is a higher frequency of pyrimidine loops in viruses infecting animals irrespective of the viruses’ genome type. This observation is confirmed by an in-depth analysis of the Herpesviridae family of viruses, which showed a distinctive accumulation of thermally stable C-looped quadruplexes in viruses infecting high-order vertebrates. The occurrence of viral C-looped G4s, which carry binding sites for host transcription factors, as well as the high prevalence of viral TTA-looped G4s, which are identical to vertebrate telomeric motifs, provide concrete examples of how PQSs may help viruses impinge upon, and benefit from, host functions. More generally, these observations suggest a co-evolution of virus and host PQSs, thus underscoring the potential functional significance of G4s.

## 1. Introduction

G-quadruplexes (G4s) are alternative DNA or RNA secondary structures formed by the stacking of planar arrangements of guanine residues, further stabilized by monovalent cations [1]. The importance of quadruplex-forming sequences as regulatory elements has been supported by extensive evidence in eukaryotic cells [2,3,4]. Putative quadruplex-forming sequences (PQSs) are prevalent in numerous genomes [5,6] and have been implicated in key genomic functions, such as transcription regulation, replication, repair, and telomere maintenance reviewed in [7,8,9]. Typically, the consensus sequence motif G_3+_N_1–7_G_3+_N_1–7_G_3+_N_1–7_G_3+_ has been used to identify potential PQSs [5,10]. This has led to an estimate of over 400,000 PQSs in the human reference genome, with a median density of 0.5 motif per kbp. In other eukaryotes and in bacteria, the density of G4 motifs is highly variable (2.5 to >0.1 motifs per kbp) [6].

PQSs are also present in viral genomes [11,12], and emerging evidence suggests that they can be implicated in viral replication and recombination, in the regulation of virulence via gene expression control [13,14], and in key steps in the viral cycles [15]. The presence of putative G4 sequences has been reported in various viral genomes, such as the human immunodeficiency virus (HIV-1) [16,17,18,19,20], the Epstein–Barr virus (EBV) [21,22], or papillomaviruses (HPV) [23,24,25]. In particular, the Epstein–Barr virus encodes the genome replication and maintenance protein EBNA1 that binds G-rich sequences to recruit the replication complex [21]. The herpes simplex virus 1 (HSV-1) genome displays multiple clusters of repeated sequences forming very stable quadruplexes that are involved in viral DNA replication [26]. The HIV-1 promoter contains a highly conserved G-rich region able to fold into a G4 structure [19] and is involved in the regulation of viral replication [18]. The presence of highly conserved PQSs able to potentially form intermolecular G4s has been reported in several human herpesvirus packaging signals [27], as well as in HIV-1 [28], further highlighting the biological role of viral G4s. In addition, several DNA aptamers (short synthetic single-stranded oligonucleotides that specifically bind to various molecular targets) containing G4-forming sequences were found to have antiviral activity [29,30] and have been used as diagnostic tools to detect viruses [11].

Within a quadruplex, the length of the G-tracts as well as the length and the base composition of the loops are critical to determine the conformation of the G4s and their stability [31,32,33,34,35,36]. Remarkably, it has been observed that most quadruplex-forming sequences found in gene promoters contain at least one single-nucleotide loop [37,38,39,40,41]. Genome-wide, our analyses across numerous eukaryotes outlined a striking enrichment of single-nucleotide loop G4s and further revealed a prominent trend favoring pyrimidine nucleotides in these loops as well as the accumulation of G_15+_ sequences in plants and invertebrates [36]. Whether these divergent evolutionary trends reflect differential biases in mutagenesis and DNA repair mechanisms from species to species and/or are the result of functional selection remains an open question.

Given that viruses utilize the hosts’ cellular machineries for replication and transcription, especially in large DNA viruses [42], we wished to examine whether the composition of G4 motifs in the viral genomes could be correlated to that of their hosts. To address this question, we identified and analyzed all G4 motifs (size, loop length, and nucleotide compositions) present in the currently available 7370 viral genome assemblies, which include seven viral taxa infecting five evolutionary distant groups of eukaryotic hosts: vertebrates (including *Homo sapiens*), invertebrates, protozoa, fungi, and plants. Here, we provide a large comparative view of the quadruplex motif loop content at nucleotide-level resolution, with particular focus on the Herpesviridae family, ubiquitous large dsDNA (linear double-stranded DNA) viruses that are amongst the best characterized host-adapted viruses.

## 2. Results and Discussion

### 2.1. G-Quadruplex Metrics in Viral Genomes

**Figure 1:**
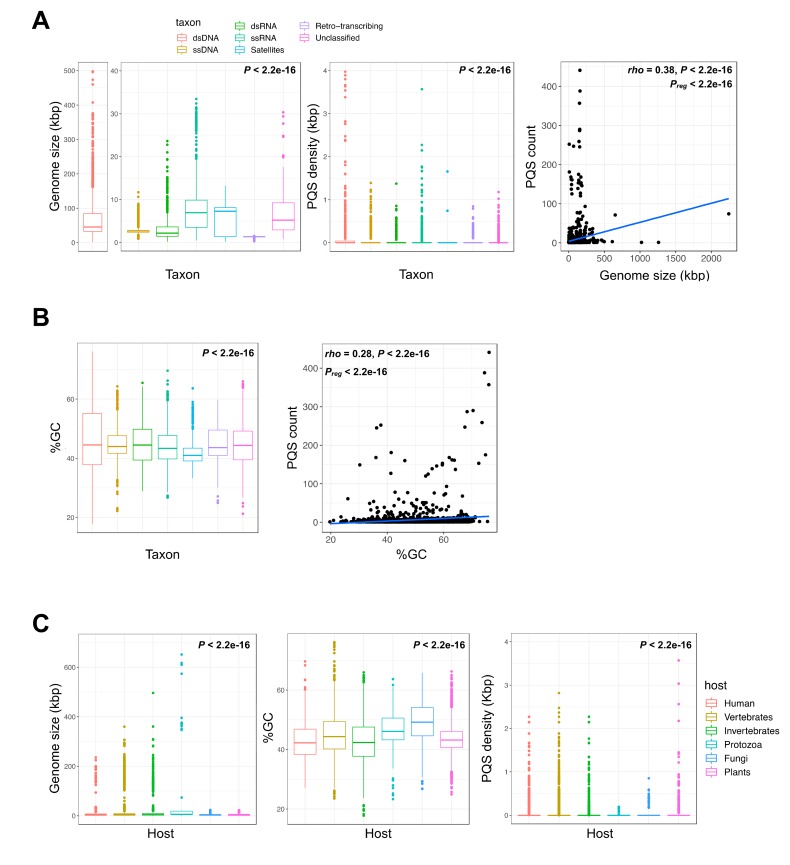
Genome metrics and quadruplex motif content of viral genomes. (**A**) From left to right, genome size (in kilo base pairs, kbp), putative quadruplex sequence (PQS) density (number of motifs found per kbp), and relationship between PQS content and genome size for different viral taxa. (**B**) GC content and relationship between PQS content and GC content for different viral taxa. (**C**) From left to right, genome size, GC content, and PQS density for different eukaryote host groups. For panels A through C, differences in average size, GC, and density values were assessed using Kruskal–Wallis rank sum tests and pairwise Wilcoxon rank-sum tests. Spearman correlation coefficients and their statistical significance are provided at the top of the scatterplots. Regression lines are shown in blue (P_reg_, linear regression significance).

To analyze the G4 motifs in a large panel of viruses, we retrieved the 7370 viral genome assemblies from the viruSITE [43] database. This database comprises all curated virus genomes available in the NCBI Reference Sequence Database (RefSeq), categorized into seven viral taxa: dsDNA, ssDNA, dsRNA, ssRNA, satellites, as well as retro-transcribing or unclassified viruses (Figure 1, panels A,B). These viruses infect a broad range of eukaryotic hosts (Figure 1C).

First, we analyzed several viral genome metrics: genome size (kilo base pairs, kbp), which varies from 0.2 kbp to over 2400 kbp; GC content (%), which varies from 17.8% to 76.1%; and PQS densities (PQS/kbp), that allow to compare the quadruplex content of each assembly independently of the genome lengths, as well as their presence on the positive (G-rich) or negative (C-rich) strand (Materials and Methods). To identify the G4 motifs, we searched the canonical G_3+_N_1–7_G_3+_N_1–7_G_3+_N_1–7_G_3+_ sequences by regular expression matching (Materials and Methods), as previously described for eukaryotic genomes [5,10,36]. All the identified quadruplex sequences are individually reported along with their coordinates in Table S1 (Supplementary Materials), which we propose as a resource. Finally, we performed virus–host analyses, classifying the eukaryote hosts into vertebrate or invertebrate animals, protozoa, plants, and fungi species. Unless otherwise mentioned, the group of vertebrates includes *Homo sapiens*.

The overall viral PQS metrics, classified by viral taxa or host group, are summarized in Table 1 and Table 2, respectively. Not surprisingly, the total number of quadruplexes depends on the viral genome size, albeit it displays an uneven density and moderate correlation (Spearman’s *rh*o = 0.38, non-parametric test for association between paired samples *p* < 2.2 × 10^−16^; Figure 1A). Of note, the dsDNA viruses, which greatly vary in genome size, show the highest densities of G4 sequences in the retrieved viral genomes (with an average of 0.08 ± 0.79 PQS/kbp; Table 1). Among them, the Herpesviridae family, further analyzed hereafter, exhibits the highest PQS content: we found a total of 6735 motifs, with an average density of 0.45 ± 0.60 PQS/kbp and up to 2.8 PQS/kbp in the Papiine alpha herpesvirus 2 (Table S2). In the remaining viral taxa, that include fewer G4 motifs and shorter viral genomes, the density of PQSs is not negligible since ssDNA, ssRNA, and retro-transcribing (RT) viruses carry 0.04 ± 0.18 to 0.07 ± 0.16 PQS/kbp (Table 1). However, as previously observed for human viruses [12], the viral genomes are in general relatively G4-poor, with a PQS density of <0.1 motif per kbp (Figure 1A). Nevertheless, PQSs are not less frequent than in zebrafish (0.019 G4/kbp), lower-order groupings of eukaryotes (e.g., 0.02 G4/kbp in *Caenorhabditis elegans*, 0.002 G4/kbp in *Plasmodium*), or bacteria (0.001 to 0.02 G4/kbp) (Table 3).

We observed that giant viruses infecting protozoan hosts are relatively enriched for G4 sequences, reaching 181 PQSs in the 2,473,870 bp (0.07 PQS/kb) *Pandoravirus salinus* genome. Intriguingly, the Mimiviridae viruses, which exhibit a low GC content (≈28%), are exceptionally G4-poor with only three G4 motifs found in the Mimivirus terra2 assembly (1,168,989 bp; 0.003 PQS/kbp). Globally, there is a significant positive correlation between the PQS and GC content, although the relationship is weak (Spearman’s *rho* = 0.28, non-parametric test for association between paired samples *P* < 2.2 × 10^−16^; Figure 1B) and the median GC content of the various viral taxa are rather similar (44% ± 8), albeit with large variations within each taxon (Figure 1B). Thus, the impact of the GC content on the probability to create a G4 motif is not strong, suggesting that at least a fraction of these quadruplex sequences may be maintained under positive selection.

Finally, in our broad viral set, we detected a significant enrichment for PQSs in the negative (C-rich) strand of dsDNA, ssRNA, RT, and unclassified viruses but not for the ssDNA virus (Figure S1A, Supplementary Materials). The overall significance of this strand bias is likely diverse and complex. Two recent functional studies suggested different and non-exclusive explanations. On one hand, Jaubert and colleagues showed that the formation of quadruplexes in the negative RNA strand of the hepatitis C virus is associated with impaired RNA synthesis [45]. On the other hand, Ding and colleagues outlined the strong bias for quadruplex sequences in the negative strand flanking the transcription start sites (TSS) in microorganisms (*Deinococcales* and *Thermales* bacterial orders), and correlated it with oxidation-dependent regulation of transcription [46].

The distribution of genome size, PQS density, GC content, and strand biases of the viruses with respect to their various hosts are reported in Figure 1C. Table 2 shows a balanced representation of the various host groups, after assessing an equivalent number of assemblies for vertebrate (2769), invertebrate (2930), and plant (2484) hosts. Within these large groups, several species are well represented. For example, in the large vertebrate group, there are numerous viruses infecting Cercopithecidae (58), Suidae (120), Bovidae (112), Pteropodidae (158), and rats (289), and over 40 avian viruses (Table S1, Supplementary Materials). Although the data were scarcer for protozoa and fungi (61 and 262 viruses, respectively), the viral genome sizes are longer in protozoa and very short in fungi and plants (Figure 1C). The PQS density is 2- to 3-fold higher in viruses infecting vertebrate hosts than any other host (Table 3). Viruses infecting plants seem particularly G4-poor (only 261 PQSs found in over 2000 genomes), with the exception of many mosaic viruses (the okra, grapevine, and chayote mosaic viruses carry over five PQSs in ≈6 kbp genomes; Table S1, Supplementary Materials). When PQSs are examined versus the host taxa, a significant excess of PQSs is again observed on the viral negative (C-rich) strand for all viruses (Figure S1B, Supplementary Materials).

### 2.2. Thermodynamically Stable G4 Motifs are Enriched in Viral Genomes

**Figure 2:**
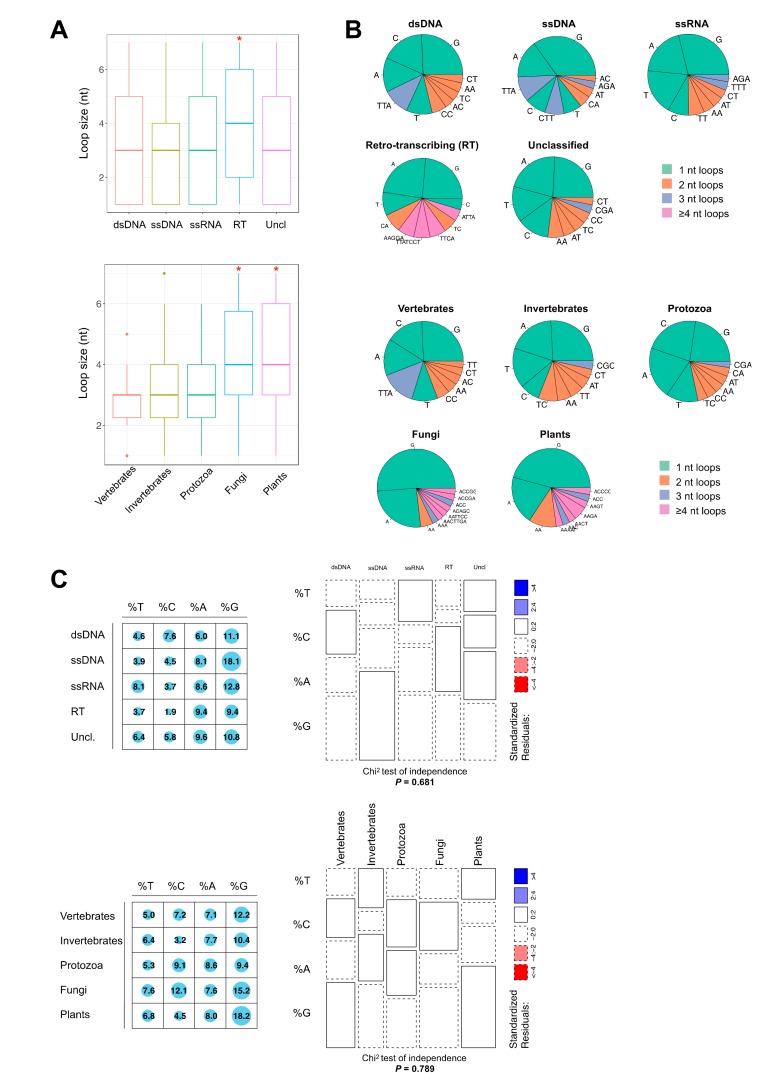
Quadruplex loop composition in viral genomes. (**A**) Boxplots show G-quadruplex (G4) motif loop size (in nucleotides) for each group. Top panel, for different viral taxa; bottom panel, for different host groups. Kruskal–Wallis rank sum test *P* = 0.0001 and *P* = 8.19 × 10^−6^ respectively; *, all pairwise Wilcoxon rank-sum tests adj*P* < 0.01. (**B**) Frequencies of 1–7 nucleotide loops, irrespective of their position within the G4 motif. Top panel, five taxa in which a significant number of G4 sequences were found: dsDNA viruses (n = 2758 assemblies, 11,315 PQSs), ssDNA viruses (n = 988 assemblies, 102 PQSs), ssRNA viruses (n = 1784 assemblies, 553 PQSs), Retro-transcribing viruses (n = 153 assemblies, 85 PQSs), and Unclassified viruses (n = 1217 assemblies, 253 PQSs). Bottom panel, five eukaryotic host taxa used in the analyses: vertebrates (n = 2,769 assemblies, 7945 PQSs), invertebrates (n = 2930 assemblies, 1410 PQSs), protozoa (n = 61 assemblies, 618 PQSs), fungi (n = 292 assemblies, 41 PQSs), and plants (n = 2484 assemblies, 261 PQSs). (**C**) Top panel and from left to right, graphical matrix where each cell contains a dot whose size reflects the relative magnitude of nucleotide proportions by viral taxa and mosaic plot of the contingency table used to perform a chi-square independence test (non-significant, *P* = 0.681); bottom panel and for left panel to right, similar for each host group (chi-square independence test non-significant, *P* = 0.789).

To examine in more detail the nature of the quadruplex motifs, we inspected loop lengths and nucleotide compositions (irrespective of their position) for the dsDNA (11,315 PQSs), ssDNA (102 PQSs), ssRNA (553 PQSs), RT (85 PQSs), and bulk unclassified (206 PQSs) viruses. Unfortunately, the few PQSs identified in the dsRNA and satellite viruses were insufficient to pursue such in-depth analyses. As shown in Figure 2A,B, the PQS loop features differ. In the most represented vertebrate-infecting viruses, we counted 2555 different loop sequences, but 393 in the invertebrates, 648 in the protozoa, and only 66 and 52 in the fungus- and plant-infecting viruses, respectively. The median loop size is 3 nucleotides across all viral taxa, with the exception of retro-transcribing (RT) viruses which carry slightly larger loops (4 nt, all pairwise Wilcoxon rank-sum tests adj*P* < 0.01; upper panel Figure 2A). Fungus and plants viruses also bear significantly larger loop size, frequently reaching 6–7 nt (Figure 2A), with a median value of 4 nt (all pairwise Wilcoxon rank-sum tests adj*P* < 0.01 except Fungi-Plants adj*P* = 0.542; lower panel Figure 2A). Furthermore, these analyses indicated that retro-transcribing, plant-infecting, and fungus-infecting viruses also show more heterogenous loop distributions than other groups (Figure 2B). Intriguingly, as previously observed in a large spectrum of eukaryotic species [36], quadruplex motifs with single-nucleotide loops are predominant both when scanned by viral taxon (upper panel, Figure 2B) or host group (lower panel, Figure 2B). Quantitatively, the single A/T/C or G loops account for 34% of all loops when scanning by viral taxon (median value; Figure 2C) or 31% when searching the hosts genomes (median value; Figure 2C).

Thus, based on the rather short length of the loops, there is an overall bias for the most thermodynamically stable G4 motifs in viral genomes, similar to other genomes [31,34,35,36]. However, considering this large classification level, there is no significant difference in the distribution of single-nucleotide loop motifs between viral taxa (chi-square independence test non-significant, *P* = 0.681; upper panel Figure 2C) nor host group (chi-square independence test non-significant, *P* = 0.789; lower panel Figure 2C). Nevertheless, there is a striking resemblance in the distribution and frequency of the loop nucleotides when comparing dsDNA viruses and their vertebrate hosts: 9 out of the 10 most frequent loops are the same in both sets (G, C, A, TTA, T, CC, AA, AC, and CT loops), and are distributed in similar proportions (Figure 2B). Among dsDNA viruses, herpesviruses are particularly enriched for short-looped quadruplex motifs, which account for 35% of all PQSs (2,355 G4-L1-3 motifs, that is, the loop size is comprised between 1 and 3 nt (Table S2, Supplementary Materials).

### 2.3. The PQS Loop Composition Within the Herpesviridae Family of Viruses and Their Host are Correlated

**Figure 3:**
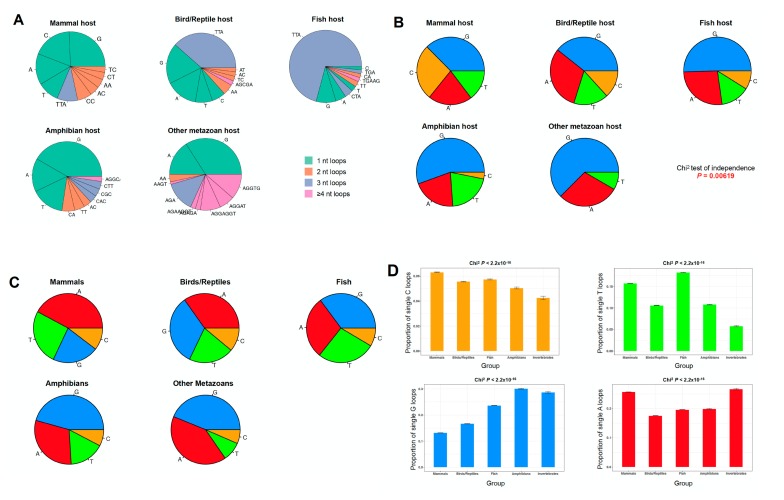
PQS loop content in Herpesviridae viruses and in various host animal genomes. (**A**) Most frequent N_1-7_ loops by host group. Mammals, n = 65 viruses; Birds/Reptiles, n = 11 viruses; Fish, n = 6 viruses; Amphibians, n = 4 viruses; and Invertebrates, n = 7 viruses. (**B**) Single-nucleotide loop frequencies by host group. Blue, G loops; red, A loops; orange, C loops; and green, T loops, irrespective of their positions with the G4 sequence. Chi-square independence tests were used to evaluate the significance of the association between loop proportions and host group. (**C**) Single-nucleotide loop frequencies in 52 eukaryote genomes. Mammals, n = 18 genomes; Birds/Reptiles, n = 9 genomes; Fish, n = 8 genomes; Amphibians, n = 3 genomes; and Invertebrates, n = 9 genomes. (**D**) Proportion of single C (orange, top panel), T (green, top panel), G (blue, bottom panel), or A (red, bottom panel) loops by eukaryote groups. Bars indicate the upper and lower bounds of the 95% confidence intervals. Chi-square independence tests were used to evaluate the significance of the association between loop proportions and group. All pairwise nominal independence adjusted *P*-values <0.05.

Since herpesviruses infect different animal hosts, including mammals, birds, reptiles, fish, amphibians, and invertebrate animals [47], we examined in more detail the relationship between the viral and host PQSs. For this purpose, we retrieved all the available herpesvirus assemblies (n = 93 genomes). These include viruses in 65 mammals, 11 birds or reptiles, 6 fish, 4 amphibians, and 7 invertebrates (Table S2, Supplementary Materials). The PQS genome metrics for these 93 assemblies are reported in Figure S3A (Supplementary Materials). While the PQS occurrence and GC content remain strongly correlated (Spearman’s *rho* = 0.73, non-parametric test for association between paired samples *P* = 1.2 × 10^−15^; Figure S3B, Supplementary Materials), we found no linear relationship between PQS content and genome size within this subset of viruses (Spearman’s *rho* = 0.10, *P* = 0.38; Figure S3B, Supplementary Materials). However, there are significant differences in the nucleotide loop composition depending on the animal host (Figure 3A). Furthermore, single-nucleotide loops are unevenly distributed when looking at different host species (chi-square independence test *P* = 0.00619; Figure 3B). Single G loops are largely prevalent in herpesviruses infecting fish, amphibians, and invertebrates (50%, 56%, and 63% of all single-nucleotide loops, respectively). In addition, single C loops were undetected in the latter, and was marginal in the first two groups (9% in fish hosts and 3% in amphibian hosts). To a lesser extent, single T loops are more frequent in viruses infecting vertebrate hosts (on average 16% of all single-nucleotide loops) than invertebrates (8% of all single-nucleotide loops). Interestingly, the observed trends recapitulate the same existent biases in the host species (Figure 3, panels C,D): for instance, the analysis of the loop composition of short-looped G4 motifs in 52 eukaryote genomes (see Materials and Methods; Figure S4, Supplementary Materials) shows an enrichment for single G loops in amphibians and invertebrates as well as an accumulation of single T and C loops in mammals, birds, and reptiles, with the frequency of C-rich loops reaching its highest levels in viruses infecting mammals. Overall, we observed an excess of the frequency of PQSs with single pyrimidine loops in herpesviruses infecting vertebrate hosts. Interestingly, in vitro, these motifs fold into the most stable G4 structures [35,36].

**Figure 4:**
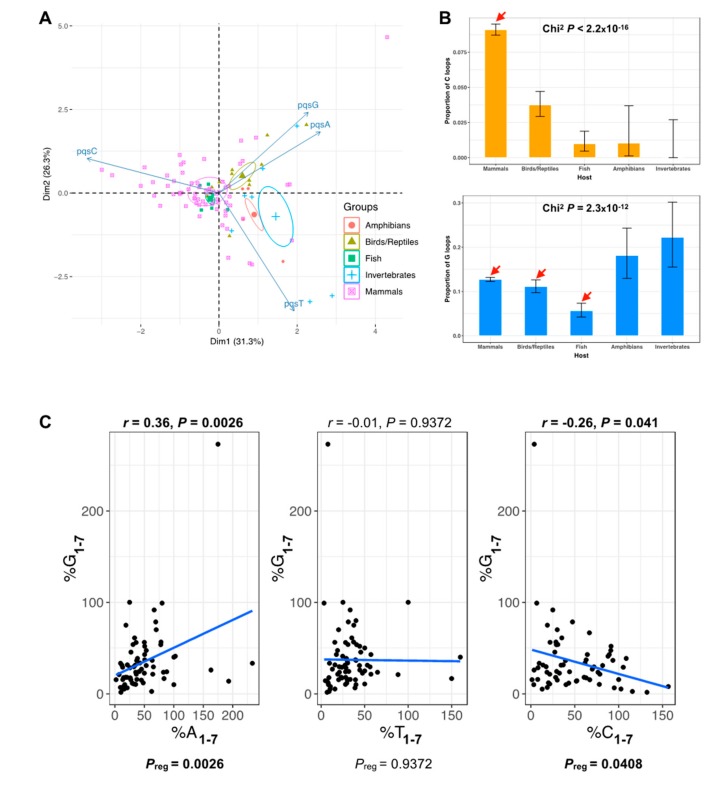
Herpesviridae viruses infecting mammalian hosts carry preferentially C-looped motifs. (**A**) Principal component analysis (PCA) performed on loop composition information (variables: PQS^A^, PQS^T^, PQS^C^, PQS^G^) for 93 herpesviruses, without using host group information. Ellipses indicate barycenters (weighted center of mass) for each host group. (**B**) Proportion of single C (orange, top panel) or G (blue, bottom panel) loops by host group. Bars indicate the upper and lower bounds of the 95% confidence intervals. Chi-square independence tests were used to evaluate the significance of the association between loop proportions and host group. Red arrows indicate groups with pairwise nominal independence adjusted *P*-values <0.05. (**C**) Relationship between PQS^A^ (PQS^T^ or PQS^C^) motif and PQS^G^ motif contents. Pearson correlation coefficients are reported on each graph. Blue lines show linear regressions (P_reg_, linear regression significance).

To further analyze this particular trend, we next performed an unsupervised classification, by principal component analysis, of herpesvirus assemblies based on loop composition information (Figure 4A; Materials and Methods). The first two principal components account for a restricted fraction of the sample’s variance (≈58%); however, quadruplex loop composition information allowed to discriminate between viruses infecting higher-order vertebrate or invertebrate hosts, mainly driven by differences in PQS^C^ content (Figure 4A). Indeed, we clearly observe two opposite trends in single-nucleotide loop distributions, with a significantly higher amount of single C loops in mammal-infecting viruses and, conversely, significantly more polyG sequences in invertebrate animals and amphibians (Figure 4B). These trends can be extended to the comparison of quadruplexes carrying identical loops of any size, as there is a negative correlation between G_1–7_ loop content and C_1–7_ loop content (Pearson’s *r* = −0.36; Figure 4C).

### 2.4. G4 Motifs in Viral Genomes Overlap Hosts’ Transcription Factor Binding Sites

**Figure 5:**
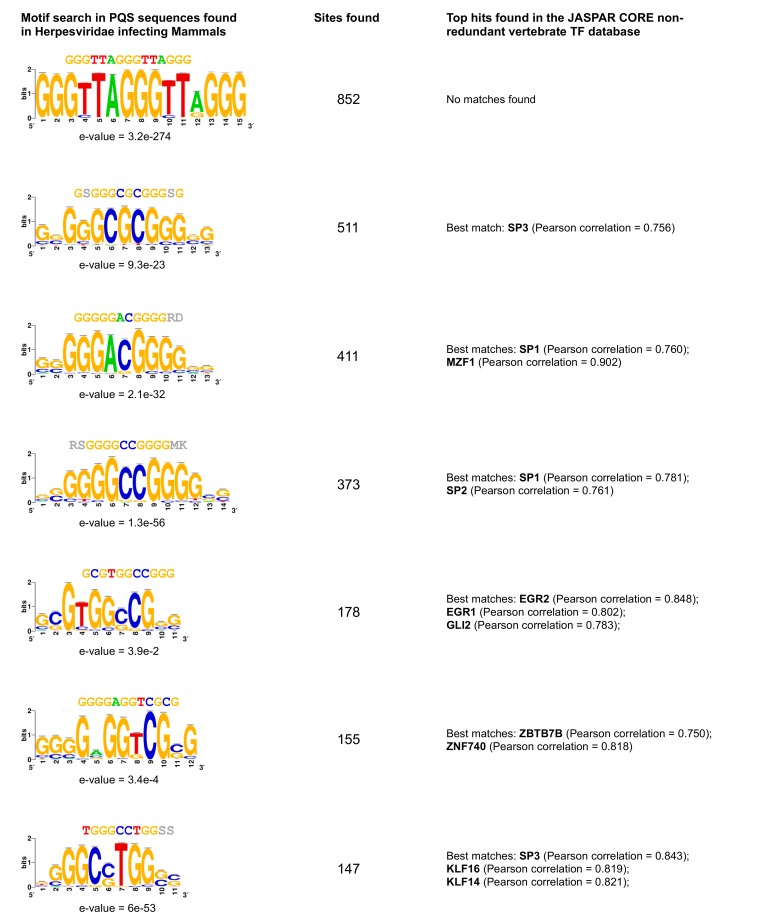
Consensus motif discovery in the quadruplex sequences found in mammalian herpesviruses. Top motifs found within the 5767 PQSs present in the 65 assemblies of herpesviruses infecting mammalian hosts. E-values are specified next to each sequence logo (the relative sizes of the letters indicate their frequency in the sequences and the total height of the letters depicts the information content of the position, in bits). The “Sites found” column indicates the number of times each particular motif was found in all the sequences.

The prevalence of C-rich PQSs found in mammalian herpesviruses raises the question whether their functional role(s) are related to their potential to form a G4 structure, to the fact that they constitute a one-dimension target sequence for the binding of host’s transcription factors, or to both. Consistent with this view, we observed that 6 of the 10 most common viral PQS motifs matched several vertebrate transcription factor sites, notably SP1 and SP3 (Figure 5). Although C-rich loops appear relatively depleted in mammalian genomes, the annotation of G4 motifs, especially in humans, shows that quadruplexes with C-rich loops (and particularly single C loops) are highly enriched at gene promoters (Figure S5, Supplementary Materials). In silico analyses of the human genome have already revealed that G4 motifs often overlap with zinc-finger transcription factor binding sites (Figure S5, Supplementary Materials), such as SP1 [48]. This observation, together with the high frequency of C-rich loops in herpesviruses infecting mammals, supports the view that the herpesviruses hijack host transcription factors during the virus life cycle. Interestingly, human transcription factors that bind to these motifs also play roles in viral infection processes (SP factors [49]; EGR2 [50]). Moreover, it has been suggested that virus-associated PQSs can be recognized by human G4-binding proteins [51], which can participate in replication associated processes.

### 2.5. High Prevalence of Telomere-Like PQSs across Herpesviridae Infecting Vertebrates

Another remarkable feature of the most frequent PQS loop composition is the excess of TTA loops that are frequent in viruses infecting mammals, birds, reptiles, and fish (Figure 3A), a loop composition that is also frequent in their hosts (Figure S4, Supplementary Materials). Of note, the TTA triplet is part of the TTAGGG telomere sequence in all vertebrates, able to form telomeric DNA G-quadruplexes [52]. It is also remarkable that the human herpesvirus 6A and 6B can integrate their linear genome into the telomeres of infected cells [53,54]. Thus, the presence/enrichment of these TTA-looped G4 sequences close to the viral genome extremities, their requirement for efficient virus integration [55], and the observation that G4 ligands can interfere with virus integration [54] point to the role played by these sequences in this crucial process that ensures virus maintenance in latently infected human cells. This potential for telomere integration is not exclusive to human herpesviruses, since it has been also described in the oncogenic Marek’s disease alpha-herpesvirus, which infects chicken lymphocytes [56]. This particular virus also carries TTA-looped G4 repeats at the ends of its linear genome, and its pathogenicity partially depends on the efficiency of telomere integration [56]. Altogether, these observations suggest that the broad presence of viral TTA-looped quadruplex sequences might be functionally and evolutionary related to the telomere biology of the hosts. If so, the presence of viral G4s with TTA loops may help predict their integration potential.

## 3. Materials and Methods

### 3.1. Genome Assembly Retrieval

The full-length sequences were retrieved from the viruSITE resource [43]. A total of 7370 sequences were analyzed, including exclusively curated assemblies extracted from numerous resources (NCBI RefSeq, UniProtKB, GO, ViralZone, PubMed). Assemblies were classified either by virus taxonomy (dsDNA viruses, dsRNA viruses, retro-transcribing viruses, satellites, ssDNA viruses, ssRNA viruses, unclassified viruses, virus-associated RNAs) or by host group (Vertebrates, Invertebrates, Protozoa, Fungi, Plants). Classification by host group was refined using the Virus-Host DB [57] resource information.

### 3.2. Genome Metrics

For each of the 7370 assemblies, genome size (in base pairs, bp) and total GC content (GC content was defined as the sum of G and C nucleotides in the respective assembly) were evaluated using bash and Perl scripts. Spearman’s rank correlation tests were used to assess correlations between the different variables.

### 3.3. G-Quadruplex Motif Identification and Loop Composition Analysis

We define a G-quadruplex motif as a sequence with at least four runs of 3+ guanines, separated by loop sequences containing one to seven nucleotides, that may themselves be guanines. Terminal guanines were excepted as loop sequences (i.e., the motif -GGGGGATCGCTGGGG- was evaluated has having an ATCGCT loop sequence flanked by GGGGG/GGGG runs and not GATCGCTG flanked by GGGG/GGG runs). Nevertheless, single G loops were allowed in the search. We searched, by regular expression matching, for the motifs previously defined -(G{3,}[ATGC]{1,7}){3,}G{3,}- in the *fasta* file of each of the retrieved assemblies, in both DNA/RNA strands, as originally described by Huppert and Balasubramanian [5]. Then, the obtained G4 sequences were imported into the R environment [58] for further processing: PQS density was defined as the number of G4 motifs per kilo base pair (kbp), we then assessed motif strandness (present in the G-rich or in the C-rich strand), split motifs into G-runs and loop sequences, and created loop repertoires (nucleotide composition, length, number of occurrences within a given genome) by host species or by virus taxon. Chi-square independence tests were used to evaluate the significance of the association between loop proportions and host group/viral taxa, followed by pairwise nominal independence tests and Pearson’s standardized residuals calculation.

### 3.4. Putative Quadruplex Sequence Analysis in Eukaryote Genomes

We also retrieved 52 eukaryote genome assemblies from the UCSC Genome Browser portal. These included:

- 18 mammals (Minke whale *balAcu1*, Marmoset *calJac3*, Dog *canFam3*, Green monkey *chlSab2*, Kangaroo rat *dipOrd1*, Wallaby *macEug2*, Crab-eating macaque *macFas5*, Mouse lemur *micMur2*, Mouse *mm10*, Gibbon *nomLeu3*, Bushbaby *otoGar3*, Baboon *papAnu2*, Orangutan *ponAbe2*, Rhesus macaque *rheMac8*, Golden snub-nosed monkey *rhiRox1*, Squirrel monkey *saiBol1*, Tarsier *tarSyr2*, Tree shrew *tupBel1*);
- 9 birds/reptiles (American alligator *allMis1*, Chicken *galGal5*, Painted turtle *chrPic1*, Garter snake *thaSir1*, Lizard *anoCar2*, Zebra finch *taeGut2*, Medium ground finch *geoFor1*, Turkey *melGal5*, Budgerigar *melUnd1*);
- 8 fish (Elephant shark *calMil1*, Zebrafish *danRer11*, Fugu *fr3*, Stickleback *gasAcu1*, Coelacanth *latCha1*, Medaka *oryLat2*, Lamprey *petMar2*, Tetraodon *tetNig2*);
- 3 amphibians (Tibetan frog *nanPar1*, African clawed frog *xenLae2*, *Xenopus tropicalis xenTro7*);
- 9 invertebrates (Apis mellifera apiMel3, Caenorhabditis elegans ce11, Caenorhabditis japonica caeJap1, Caenorhabditis brenneri caePb2, Caenorhabditis remanei caeRem3, Caenorhabditis briggsae cb3, Ciona intestinalis ci3, Drosophila melanogaster dm6, Pristionchus pacificus priPac1).

We searched for short-looped quadruplex sequences, by regular expression matching, -(G{3,}[ATGC]{1,3}){3,}G{3,}-, in the *fasta* file of each of the retrieved assemblies. We performed the same subsequent analyses as described for viral sequences. Canonical PQS content (-(G{3,}[ATGC]{1,7}){3,}G{3,}-) for the 12 species reported in Table 3 was retrieved from Marsico et al. [44] and densities were calculated, as before, counting the number of PQSs per kilo base pair.

### 3.5. Loop Composition Analysis in Herpesviruses

Principal component analysis (PCA) was implemented using the FactoMineR and factoextra packages in the R environment. The analysis was performed on loop composition information (variables: PQS^A^, PQS^T^, PQS^C^, PQS^G^; where PQS^X^ is a quadruplex motif containing at least one X_1–7_ loop, X = {A,T,C, or G}), after normalizing the data matrix (variables were centered and reduced). Correlation between PQS^A^ (PQS^T^ or PQS^C^) loop content and PQS^G^ loop content was estimated by calculating Pearson correlation coefficients. Finally, pattern discovery within the quadruplex sequences found in mammalian herpesviruses or in promoter regions of the human reference genome *hg38* was performed using the RSAT software suite [59] with default settings. The set of significant motifs discovered (e-value <0.05) was compared to the JASPAR database of vertebrate non-redundant transcription factor binding motifs [60].

### 3.6. Statistics

All statistical analyses were performed in R 3.4.3 for Mac OS X [58], using the built-in stats library, and the additional pwr, rcompanion, FactoMineR and factoextra packages.

## 4. Conclusions

Here, we report the analyses of the putative G-quadruplex-forming sequence present in 7370 virus genome assemblies. We have used this exhaustive resource to examine the potential correlations with the G4 motifs of their biological host(s), taking into account the number of motifs per genome, the length of the nucleotide loops separating the G-tracks and their base composition. Remarkably, there is a predominance of single-nucleotide loop motifs in the paired viruses and animal host genomes. These G4s are the most thermodynamically stable quadruplexes, suggesting a high folding potential and stabilization in cells. We had previously observed a strong compositional bias in these sequences in eukaryotic genomes, disfavoring pyrimidine loops while resulting in the accumulation of less stable structures (carrying single A or G loops) [36]. Here, using the G4-rich Herpesviridae family of dsDNA viruses as a case study (6735 PQSs, representing 55% of all motifs found in the >7000 viral genomes), we demonstrate a correlation between G-quadruplex sequence composition in viruses and their hosts. Indeed, herpesviruses that infect mammals, birds, or fish frequently carry TTA-looped G4 sequences, the signature of telomeric G-quadruplexes in vertebrates, which can be associated with viral integration into the hosts’ genomes [55]. Although telomeric integration of herpesviruses has been shown to occur in two vertebrate hosts, the high prevalence of TTA-looped G4 in the vertebrate-related Herpesviridae family suggests that this phenomenon could occur more frequently than anticipated. Likewise, there is an accumulation of C-looped quadruplexes in viruses infecting mammals, which in turn carry significantly more such sequences than other animals. In humans, PQSs having C-rich loops, while globally depleted throughout the genome, are exceptionally enriched in promoters, where they may provide transcription factor binding sites (e.g., SP1, SP2, and other zinc-finger TFs) [48] or else promote a defined structural fold having a defined impact in transcription [61]. Thus, viral genomes are enriched with PQSs of similar loop composition to those associated with functionally relevant regions in their host species. We do not actually know if specific viral nucleotide loop patterns could have been acquired accidentally from the host as a consequence of infection, or if there are long-term virus–host co-evolution processes that influence the emergence and maintenance of particular quadruplex sequences. If so, these sequences could regulate crucial steps in the viral cycle and could represent relevant druggable structures for new anti-viral therapeutic approaches. However, pursuing further analysis of the co-evolutionary aspects hinted in this study will demand additional virus identification and sequencing, especially those infecting protozoa or plants.

## Figures and Tables

**Table 1:** Genome metrics and quadruplex sequences in seven viral taxa.

Taxon	Assemblies	Median % GC	Median Genome Size (Base Pairs)	Total PQS Count	Mean PQS Density ^1^
dsDNA	2758	44.5	45,531	11,315	0.083
dsRNA	301	44.5	2178	11	0.018
RT ^2^	153	43.6	7743	85	0.074
Satellites	227	41.0	1348	2	0.011
ssDNA	988	44.0	2707	102	0.058
ssRNA	1784	43.4	6944	553	0.036
Unclassified	1158	44.6	4492	206	0.024

^1^ Number of PQSs per kilo base pair (PQS/kbp); ^2^ RT: retro-transcribing viruses.

**Table 2:** Genome metrics and quadruplex sequences in various organisms.

Organism	Median % GC	Genome Size (Mb)	Total PQS Count ^1^	Mean PQS Density ^2^
Human	37.8	3095.69	434,272	0.140
Mouse	42.6	2730.87	327,452	0.120
Zebrafish	36.8	1371.72	25,677	0.019
*Drosophila melanogaster*	42.1	143.73	5262	0.037
*Caenorhabditis elegans*	35.4	100.29	1561	0.016
*Saccharomyces cerevisiae*	38.4	12.16	7	0.001
*Leishmania major*	59.6	32.86	7913	0.241
*Trypanosoma brucei*	46.8	35.83	635	0.018
*Plasmodium falciparum*	19.6	23.33	51	0.002
*Arabidopsis thaliana*	36.1	119.67	338	0.003
*Rhodobacter sphaeroides*	68.8	4.64	5	0.001
*E. coli*	50.8	4.6	109	0.024

^1^ PQS counts retrieved from Marsico et al. 2019 [44]; ^2^ Number of PQSs per kilo base pair (PQS/kbp).

**Table 3:** Genome metrics and quadruplex sequences classified by host group.

Host	Assemblies	Median % GC	Median Genome Size (bp)	Total PQS Count	Mean PQS Density ^1^
Vertebrates	2769	44.4	5079	7945	0.082
Human ^2^	1144	42.3	4325	1410	0.076
Invertebrates	2930	42.4	4534	442	0.024
Protozoa	61	46.1	6038	618	0.024
Fungi	292	49.2	3147	41	0.039
Plants	2484	43.2	2759	262	0.027

^1^ Number of PQSs per kilo base pair (PQS/kbp); ^2^ Host group included in the Vertebrates group.

## Supplementary Materials

The following are available online at https://www.mdpi.com/1420-3049/24/10/1942/s1, Figure S1: Quadruplex motifs found by viral genome classification and by strand, Figure S2: Quadruplex motifs found in eukaryotes, by strand, Figure S3: Genome metrics and PQS content in the Herpesviridae family of dsDNA viruses, Figure S4: G4 motif loop content in various eukaryotes, Figure S5: Consensus motif discovery in the quadruplex sequences found in promoter regions of the human genome, Table S1: G-quadruplex sequences (and coordinates) found in seven viral taxa, Table S2: Genome metrics and quadruplex sequences for 93 herpesvirus genomes.
