# Supplementary material for: Relationship Between G-Quadruplex Sequence Composition in Viruses and Their Hosts
Source: Molecules. 2019 May 20;24(10):1942. doi: 10.3390/molecules24101942 (PMC6572409; doi:10.3390/molecules24101942)
Supplement: Supplementary file 1 [file molecules-24-01942-s001.zip › Supplementary_material_Puig-Lombardi_et_al.pdf]

Supplementary material

Relationship between G-quadruplex sequence composition in viruses and their hosts

Emilia Puig Lombardi [1], Arturo Londoño-Vallejo [1,*] and Alain Nicolas [1,*]

[1] Institut Curie, PSL Research University, UMR3244 CNRS, 75248 Paris Cedex05, France

[*] Correspondence: Arturo.Londono@curie.fr (A.L.V); alain.nicolas@curie.fr (A.N.)

Contents

Figure S1: Quadruplex motifs found by viral genome classification and by strand

Figure S2: Quadruplex motifs found in eukaryotes, by strand

Figure S3: Genome metrics and PQS content in the Herpesviridae family of dsDNA viruses

Figure S4: G4 motif loop content in various eukaryotes

Figure S5: Consensus motif discovery in the quadruplex sequences found in promoters regions of the human genome

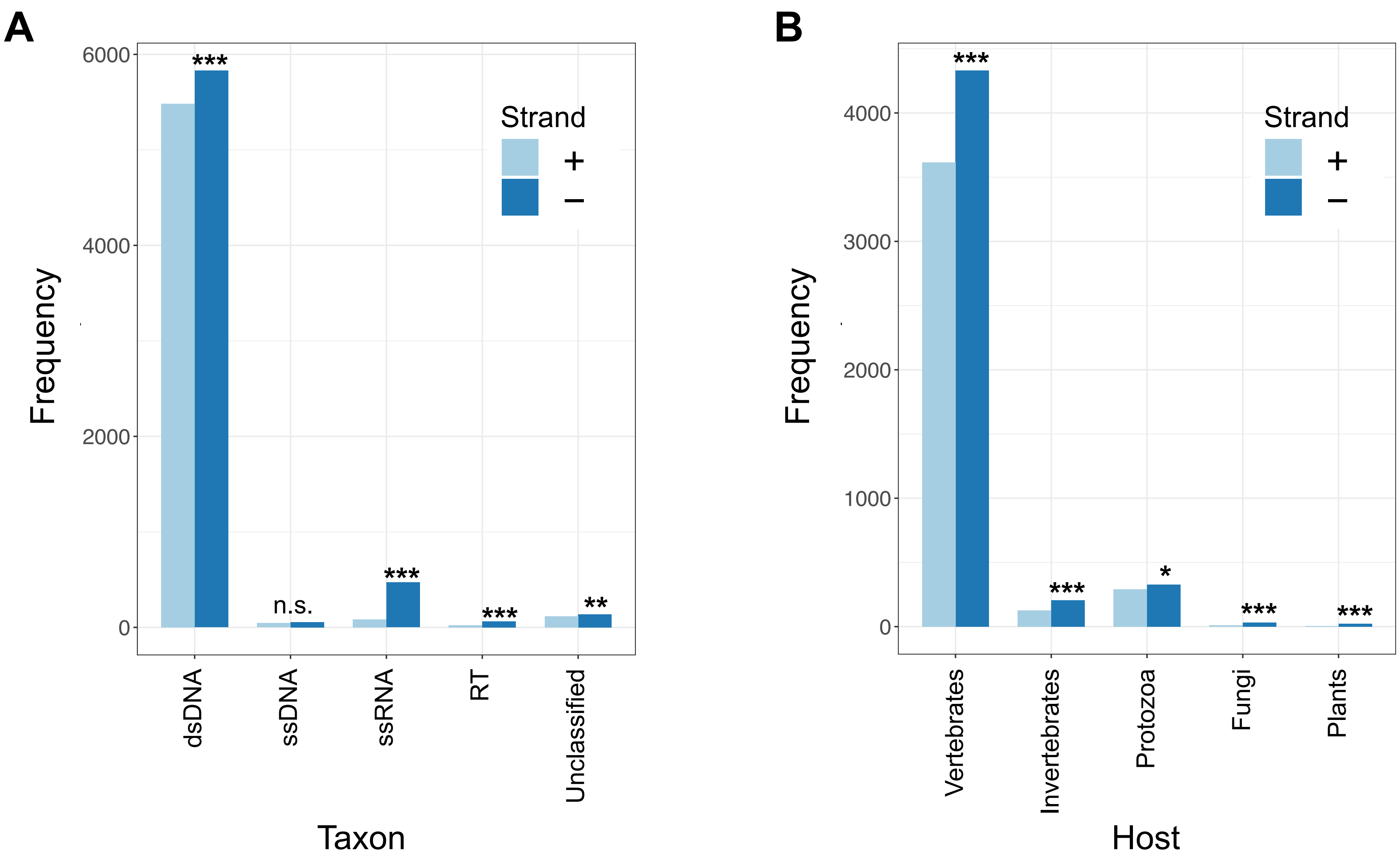

Figure S1. Quadruplex motifs found by viral genome classification and by strand. The frequencies of the detected PQS by **(A)** viral taxon or **(B)** host groups, are reported in the y-axes (median values for each group). Blue, negative strand; light blue, positive strand. ***, two-proportions z-test $P < 0.001$; **, $P < 0.01$; *, $P < 0.05$; n.s. non-significant $P > 0.05$.

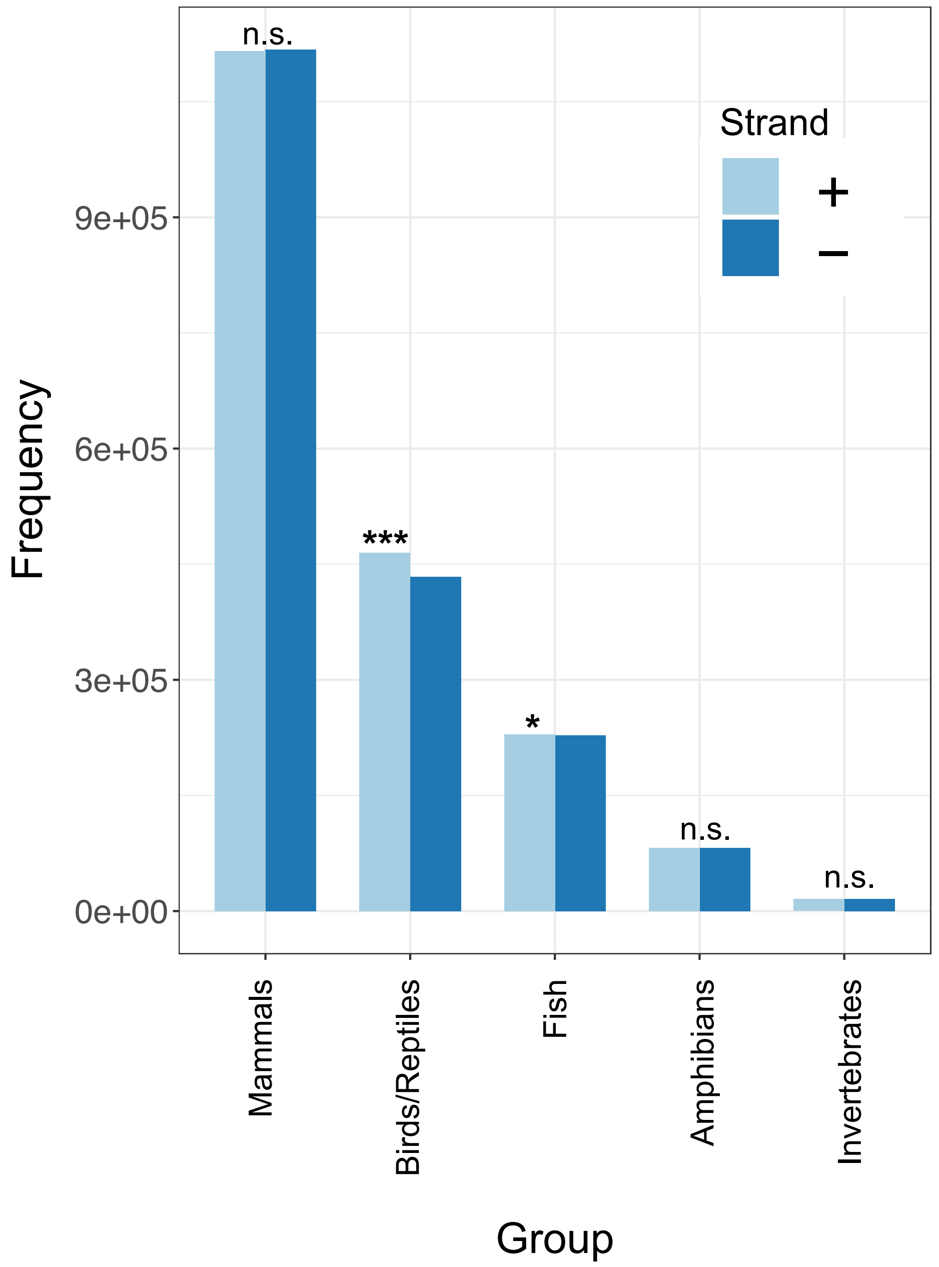

Figure S2. Quadruplex motifs found in eukaryotes, by strand. The frequencies of the detected PQS are reported in the y-axes (median values for each group). Blue, negative strand; light blue, positive strand. ***, two-proportions z-test $P < 0.001$; *, $P < 0.05$; n.s. non-significant $P > 0.05$.

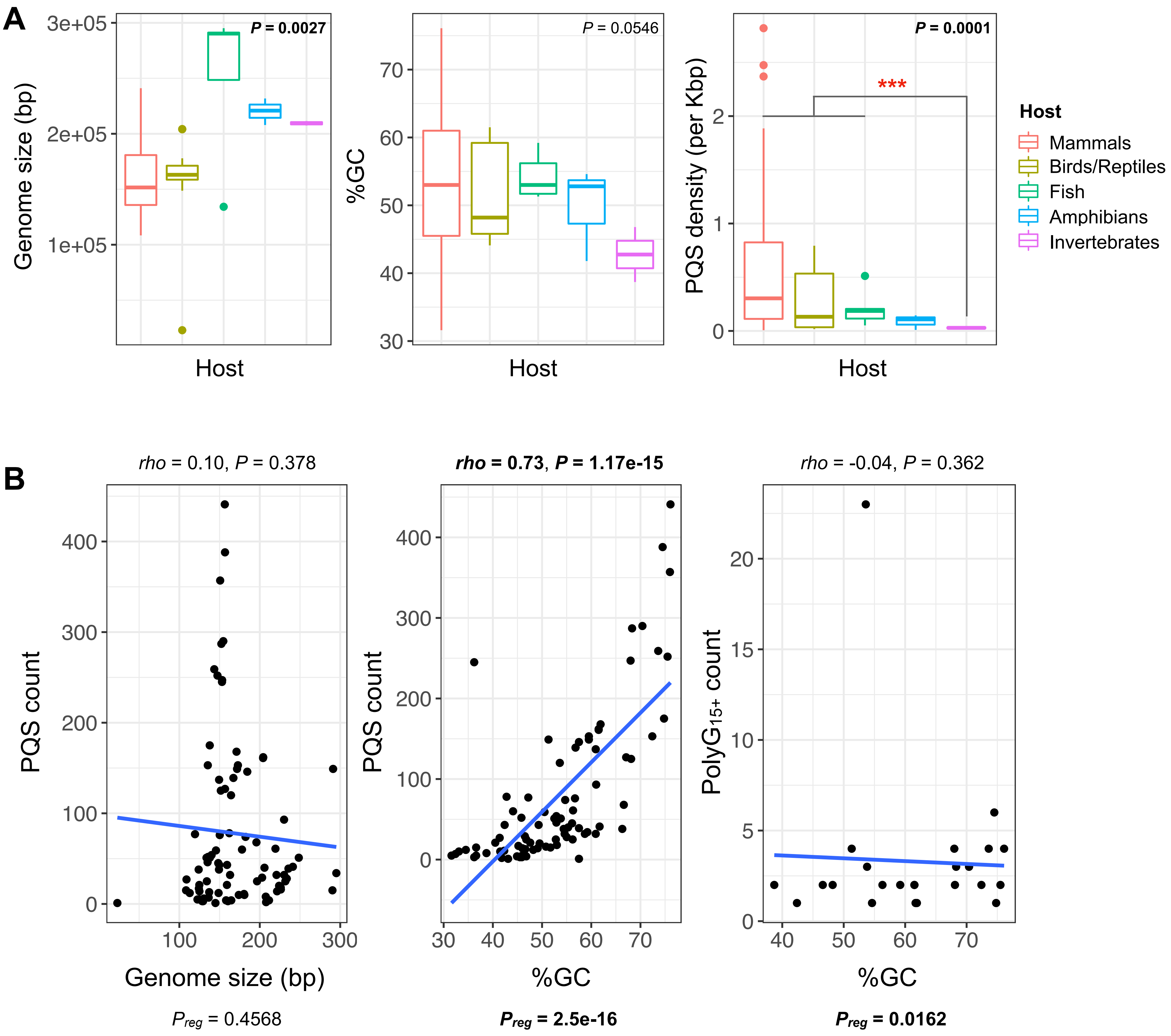

Figure S3. Genome metrics and PQS content in the *Herpesviridae* family of dsDNA viruses. **(A)** Genome size (in base pairs, bp), GC content and PQS density (number of motifs found per kilo base pair, Kbp) for different groups of eukaryotic hosts. Differences in average size, GC and density values were assessed using Kruskal-Wallis rank sum tests and pairwise Wilcoxon rank sum tests. ***, adj$P$ < 0.001. **(B)** Relationship between genome size (in kilo base pairs, Kbp) or GC content and PQS count; and between PolyG_{15+} motif content and GC content. Spearman correlation coefficients and their statistical significance are provided at the top of each panel. Regression lines are shown in blue (P_{reg}, linear regression significance).

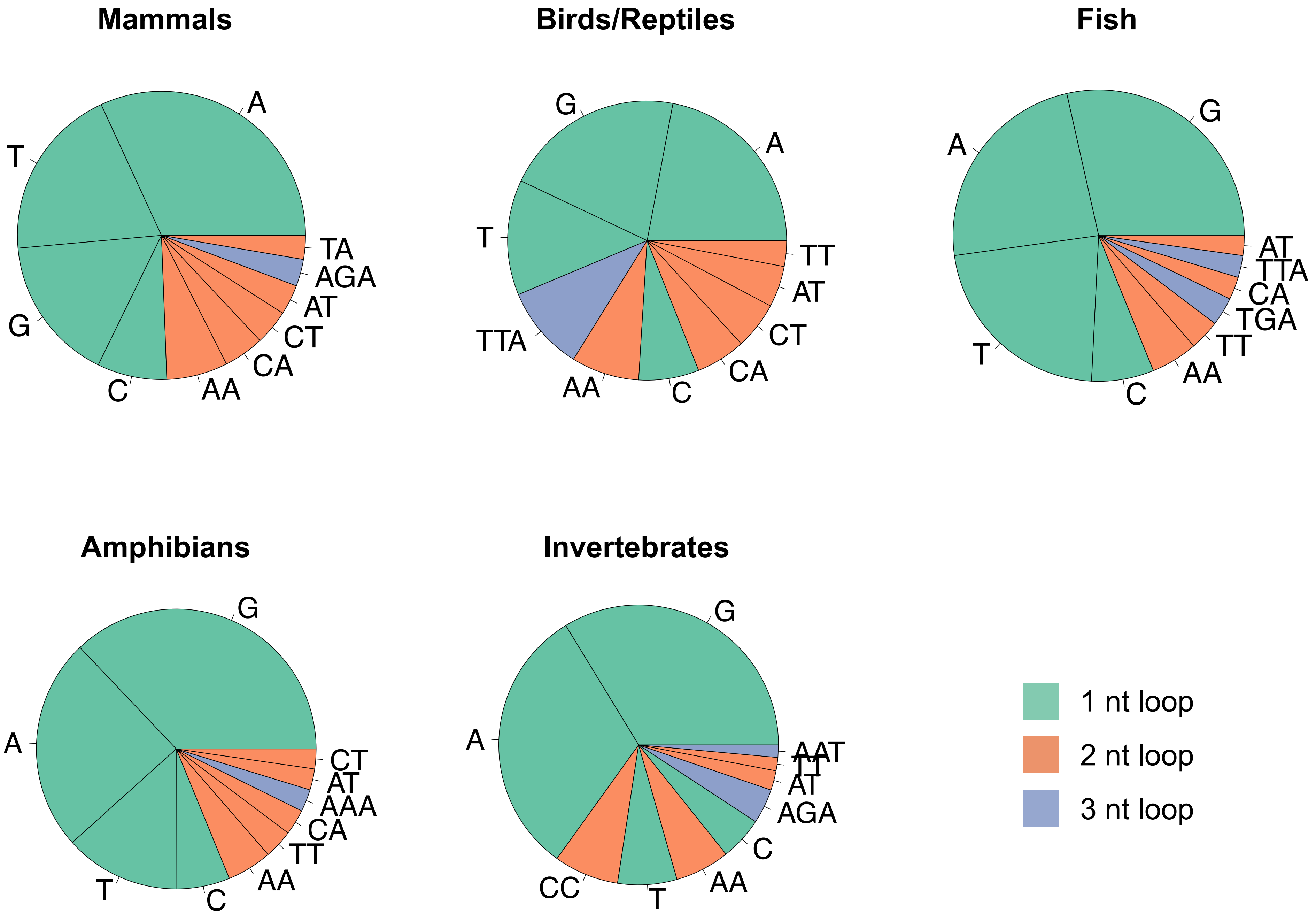

Figure S4. G4 motif loop content in various eukaryotes. Most frequent N_{1-3} loops in Mammals, n=18 genomes; Birds/Reptiles, n=9 genomes; Fish, n=8 genomes; Amphibians, n=3 genomes and Invertebrates, n=9 genomes. Green, single nucleotide loops; Orange, dinucleotide loops; Purple, trinucleotide loops.

Motif search in PQS sequences found in promoter regions (hg38)	Sites found	Top hits found in the JASPAR CORE non-redundant vertebrate TF database
GGGCGGGCGGGGGG 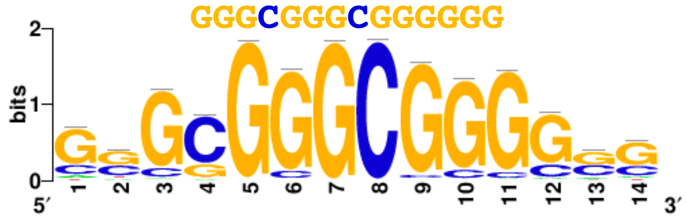 e-value = 4.9e-12	3,629	Best matches: **SP1** (Pearson correlation = 0.848); **SP2** (Pearson correlation = 0.836); **SP8** (Pearson correlation = 0.742)
GGGGCGCGGGGG 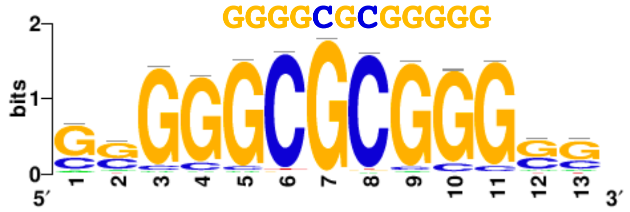 e-value = 3e-156	2,476	Best matches: **SP2** (Pearson correlation = 0.731); **SP3** (Pearson correlation = 0.751); **KLF16** (Pearson correlation = 0.733)
GMGGGGCGGGGCTGGGSK 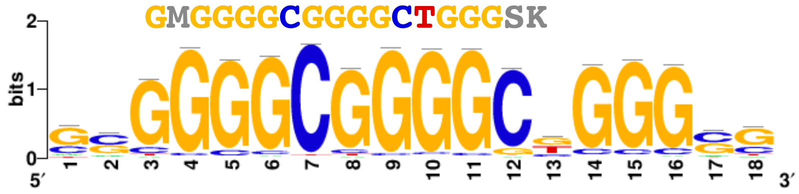 e-value = 2.9e-194	2,474	Best matches: **SP1** (Pearson correlation = 0.910); **SP2** (Pearson correlation = 0.722); **SP4** (Pearson correlation = 0.721)
SSGGGCCGGGSG 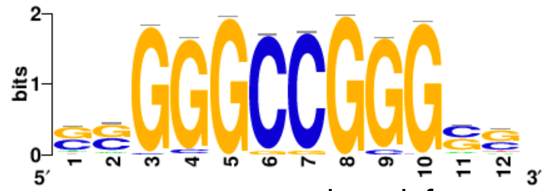 e-value = Inf	2,337	Best matches: **SP1** (Pearson correlation = 0.731); **SP2** (Pearson correlation = 0.734); **KLF5** (Pearson correlation = 0.722)
RDGGGCAGGGAD 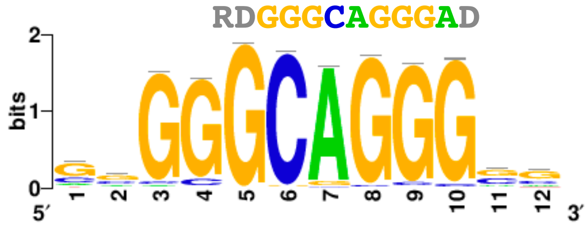 e-value = 4.4e-149	2,158	Best matches: **SP1** (Pearson correlation = 0.747); **THAP1** (Pearson correlation = 0.880); **KLF5** (Pearson correlation = 0.727)
KRGGRGGCGGGGCCTGGGVR 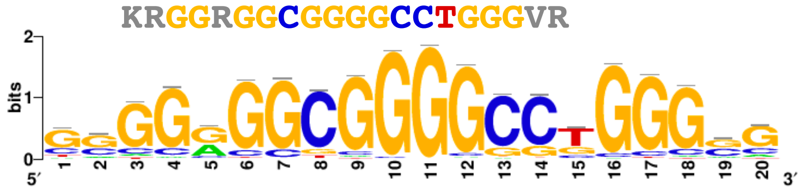 e-value = 3.2e-142	589	Best matches: **SP2** (Pearson correlation = 0.921); **SP4** (Pearson correlation = 0.728); **Zfx** (Pearson correlation = 0.732)

Figure S5. Consensus motif discovery in the quadruplex sequences found in promoters regions of the human genome. Top motifs (based on number of sites found) found within the 21,491 PQS present in the 'Promoter-TSS' annotated regions of the human reference genome *hg38*. E-values are specified next to each sequence logo (the relative sizes of the letters indicate their frequency in the sequences and the total height of the letters depicts the information content of the position, in bits). The 'sites found' column indicates the number of times each particular motif was found in all the sequences.
